# Relative Quantitation of Glycopeptides Based on Stable Isotope Labeling Using MALDI-TOF MS

**DOI:** 10.3390/molecules19079944

**Published:** 2014-07-09

**Authors:** Masaki Kurogochi, Junko Amano

**Affiliations:** Laboratory of Glycobiology, The Noguchi Institute, 1-8-1, kaga, Itabashi, Tokyo 173-0003, Japan; E-Mail: kuroko@noguchi.or.jp

**Keywords:** glycopeptides, stable isotope labeling, MALDI TOF-MS, quantitative glycopeptide profiling

## Abstract

We have developed an effective, sensitive method for quantitative glycopeptide profiling using stable isotope labeling and MALDI-TOF mass spectrometry (MS). In this study, we synthesized benzoic acid-d_0_ *N*-succinimidyl ester (BzOSu) and benzoic acid-d_5_ *N*-succinimidyl ester (d-BzOSu) as light and heavy isotope reagents for stable isotope quantification for the comparative analysis of glycopeptides. Using this approach provided enhanced ionization efficiency in both positive and negative modes by MALDI-TOF MS. These reagents were quantitatively reacted with glycopeptides from human serum IgG (hIgG) at a wide range of concentrations; the labeling efficiency of the glycopeptides showed high reproducibility and a good calibration curve was obtained. To demonstrate the practical utility of this approach, we characterized the structures of glycopeptides from hIgG and from IgG1 produced by myeloma plasma. The glycopeptides were quantitatively analyzed by mixing Bz-labeled IgG1 glycopeptides with d-Bz-labeled hIgG glycopeptides. Glycan structural identification of the hIgG glycopeptides was demonstrated by combining the highly specific recognition of endo-β-*N*-acetyl glucosaminidases from *Streptococcus pyogenes* (endoS) or from *Streptococcus pneumoniae* (endo-D) with MALDI-TOF MS analysis. The obtained data revealed the glycan profile and the ratio of glycan structural isomers containing a galactosylated extension on IgG1, IgG2 and IgG3 glycopetides.

## 1. Introduction

Glycosylation is one of the most ubiquitous and crucial processes in the posttranslational modification of proteins expressed in eukaryotic cells [1,2]. It is estimated that 50%–60% of human proteins are glycosylated [3]. The glycans play important roles in many biological phenomena, such as the stability and conformation of the protein, cellular signaling, and molecular recognition. Glycoproteomics combines proteomics and glycomics and aims to characterize the sequence, glycosylation sites, and glycan structures of the glycosylated peptides and proteins, including their microheterogeneity. Glycoproteomics has recently been used to elucidate biological functions and to explore biomarkers. The study of glycoproteomics is complicated not only because of the variety of carbohydrates that decorate proteins, but also because of the complex linkages of the glycan to the protein. Glycosylation occurs at several different amino acid residues in the protein sequence. The most common and widely studied forms are *N*-linked and *O*-linked glycosylation. *N*-linked glycans are attached to the side chain amide group of asparagine residues in a consensus Asn-X-Ser/Thr sequence, whereas *O*-linked glycans are linked to the side chain hydroxyl group of serine or threonine residues. The techniques used in glycoproteomics are becoming more sophisticated, allowing more detailed information to be obtained [4]. The development of these techniques has led to clinical translational studies and its application to several fields, in particular to protein biomarker research. These techniques are classified into three categories: glycoprotein and glycopeptide enrichment, systematic identification, and the quantification of glycopeptides in a complex sample. Several methods have been reported for glycoprotein and glycopeptide enrichment, such as lectin affinity approaches [5,6], hydrazide chemistry-based solid phase extraction approaches [7,8,9,10], boronic acid affinity approaches [11,12], and hydrophilic interaction liquid chromatography (HILIC) approaches [13,14]. Systematic identification has benefited from developments in instrumentation such as high performance fragmentation mass spectrometers (high energy collision-induced dissociation (HCD) [15], infrared multiphoton dissociation (IRMPD) [16,17], electron-capture dissociation (ECD) [16,17,18,19,20], and electron-transfer disassociation (ETD) [21,22]), as well as developments in search algorithms such as SEQUEST [23], MASCOT [24], and X! tandem [25]. A large number of annotated glycoproteins have been recorded in the UniProt database, and many MS/MS spectra are listed in the PeptideAtlas library [26]. For quantitative glycoproteomics, stable isotopic or isobaric labeling using chemical reactions (ICAT and iTRAQ) [27,28], metabolic incorporation (SILAC) [29], enzymatic reactions (^18^O labeling [30]) and MRM-MS methods using ESI-triple-quadrupole MS instruments as label-free approaches [31] have been developed. These techniques are generally based on LC-ESI-MS/MS systems with high separation performance. However, LC-ESI-MS/MS instruments are more complicated to operate than MALDI-TOF MS; for example, the ionization parameters (probe temperature, voltage, *etc.*) and LC parameters must be adjusted so that the multiply charged ions can be identified. In MALDI-TOF MS, stable isotope labeling of peptides and glycans has been reported [27,32,33], but these labeling approaches are not suitable for glycopeptides because glycopeptides exhibit low reactivity and ionization efficiency due to the large number of hydroxyl groups in carbohydrates. We previously developed an on-target labeling method for glycopeptides with pyrenyl diazomethane to enhance ionization [34]. The derivative is neutral, resulting in effective ionization and the production of positive and negative ions. However, this methodology is not suitable for comparative analysis using stable isotope labeling because the derivative is prepared on-target. This work describes how we developed a novel strategy based on stable isotope labeling allowing the highly sensitive relative quantitation of glycopeptides. The labeling of glycopeptides has been applied to cation-charged mass tags [35] bearing a quaternary phosphonium cation or a tertiary, quaternary ammonium cation, such as tris-(2,4,6-trimethoxyphenyl)-phosphonium (TMPP) derivatives, and also to a tandem mass tag (TMT) [36,37]. These approaches are similar to those used in proteomics and glycomics [38]. Stable isotope dimethyl labeling to amino groups of peptides and glycopeptides by reductive amination was often applied to LC-MS analysis [39,40,41], but it seems to be unsuitable for detection in negative mode due to a formation of tertiary ammonium ion. Although we synthesized the benzoyl-d_0_ (Bz) and benzoyl-d_5_ (d-Bz) reagents as non-charged hydrophobic mass tags, they enhanced ionization efficiency in both the positive and negative modes in MALDI-TOF MS. To demonstrate the utility of the developed approach, we characterized and quantitatively analyzed the *N*-linked glycopeptides of IgG1 from two samples: human serum IgG, and purified IgG1 produced by myeloma plasma. In addition, we applied this method to identify the substrate specificity of endo-glycosidase and obtained glycan structural identification using the highly specific recognition of endo-β-*N*-acetylglucosaminidases (endoS and endo-D). Identification was achieved using MALDI-TOF MS on crude samples. This study suggests that isotopic glycopeptide-labeling is a promising approach for quantitative glycopeptide profiling and holds significant potential for biological and clinical research.

## 2. Results and Discussion

### 2.1. Synthesis and Evaluation of Stable Isotope-Labeled Amine-Reactive Mass Tag Reagent for Glycopeptides

Stable isotope labeling mass tags have been exploited for both the relative and absolute quantification of peptides in proteomic studies [27,28,36,37].

**Scheme 1:**
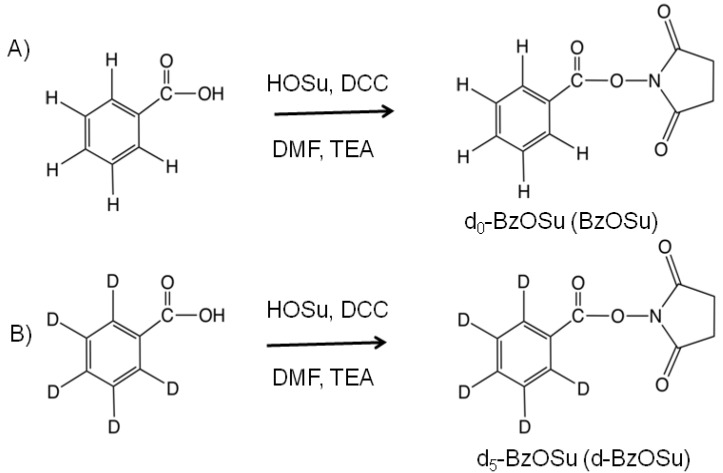
Synthesis of d_0_-BzOSu (**A**) and d_5_-BzOSu (**B**).

**Figure 1:**
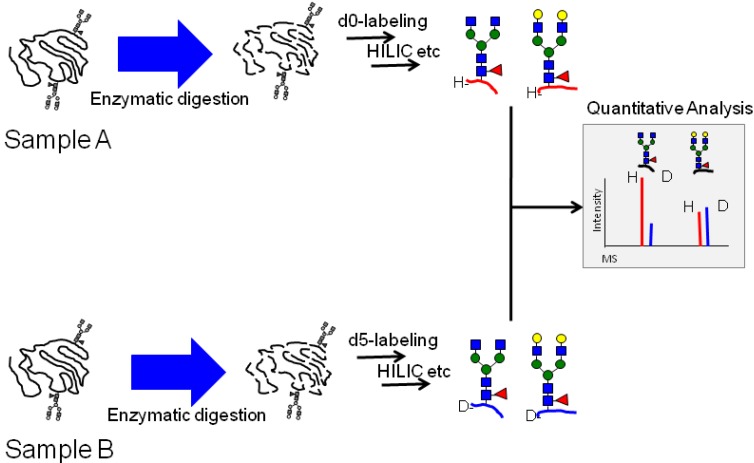
Strategy for quantitative and comparative analysis based on stable isotope labeling using MALDI TOF-MS.

We synthesized benzoic acid-d_0_ *N*-succinimidyl ester (BzOSu) and benzoic acid-d_5_ *N*-succinimidyl ester (d-BzOSu) as light and heavy isotope mass tag reagents for stable isotope-based quantification in comparative glycoproteomics (Scheme 1). These compounds were functionalized with amine-reactive groups, and reacted with amine-containing glycopeptides to form an amide bond through a mild coupling reaction. The coupled glycopeptides could be detected under both positive and negative mode conditions in MALDI-TOF MS due to their enhanced MS signal compared to unlabeled glycopeptides. Enhanced MS signals were also observed for egg yolk glycopeptides and bovine ribonuclease B glycopeptides, in a manner similar to that for hIgG glycopeptides (Supplementary Figures S1–S3). We thought that a non-charged hydrophobic mass tag would enhance ionization efficiency by preventing the aggregation of analytes, while ionic interactions and hydrophilic interactions would induce aggregation and inhibit the ionization process in both positive and negative modes on MALDI-TOF MS. Although we tested other labeling compounds, including the acetyl, naphthoyl and pyrenoyl groups, with egg yolk glycopeptides, comparative analysis by MALDI-TOF MS (Supplementary Figures S1, S4 and S5) showed that the benzoyl group was the best labeling compound for enhancing ionization efficiency. The hydrophobicity of the benzoyl group may be sufficient to inhibit the hydrophilic interaction of glycopeptides, without causing hydrophobic aggregation. We selected the benzoyl group to label glycopeptides and quantified the labeled glycopeptides by their MS spectra using isotopic light/heavy pairs (Figure 1). 

*Reagents*: HOSu: *N*-hydroxysuccinimide; TEA: triethylamine; DCC: dicyclohexylcarbodiimide; DMF: dimethylformamide).

The mass difference of 5.03 Da between the light and heavy benzoyl reagents was sufficient to separate the isotopic patterns of the glycopeptides. Although *N*-hydroxysuccinimide ester reacts with all amino groups of peptides containing the side chain ε-amino group of a lysine residue, this reagent can be incorporated at only *N*-terminal amino group of peptides after blocking the side chain ε-amino group of a lysine residue with guanidination in the same procedure as Chengjie *et al*.’s [40], for example Supplementary Figures S1,S3.

**Figure 2:**
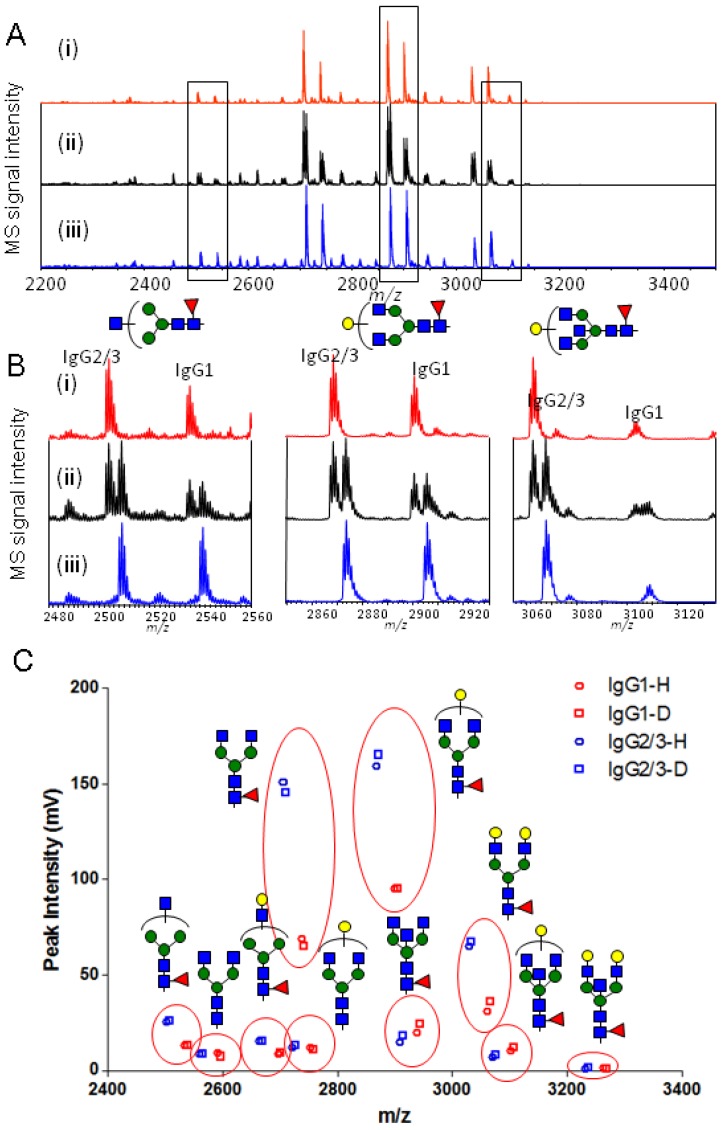
Validation of the proposed method using glycopeptides from human serum IgG. (**A**) MALDI-TOF MS spectra of (i) Bz-labeled glycopeptides (ii) an equimolar mixture of Bz- and d-Bz-labeled glycopeptides (iii) d-Bz labeled glycopeptides; (**B**) Enlarged spectra of glycopeptides containing Hex_3_HexNAc_3_dHex_1_, Hex_4_HexNAc_4_dHex_1_, Hex_4_HexNAc_5_dHex_1_; (**C**) Plots of each peak in the equimolar mixture of Bz- and d-Bz-labeled glycopeptides. (red, blue is IgG1, IgG2/3 glycopeptides; circle, square is Bz-, d-Bz-labeled glycopeptides).

Glycopeptides were prepared from normal hIgG and reacted with light and heavy isotope mass tag reagents. The labeled glycopeptides were isolated from the complex sample using the improved HILIC method reported by Wada *et al* as described in Experimental Section 3.4 [13]. Then, each obtained isotopically labeled glycopeptides and a 1:1 molar ratio mixture were measured by MALDI-TOF MS, we observed labeled glycopeptides with the mass difference of 5 Da origined from light and heavy isotope mass tag (Figure 2).

Human serum IgG is composed of four subclasses (IgG1 (66%), IgG2 (23%), IgG3 (7%) and IgG4 (4%)); the characteristics of these subclasses have been reported extensively [42,43,44]. All four subclasses have a single *N*-glycosylation site in the constant region of the heavy chain. This single *N*-glycosylation site was occupied by partially truncated biantennary glycans with or without core fucosylation or bisecting GlcNAc. Glycopeptides digested with trypsin provided four peptide moieties consisting of nine amino acids. These peptides were derived from the constant region of the heavy chains of IgG1, IgG2, IgG3 and IgG4; consequently, these glycopeptides could be identified by the peptide molecular weight. In this study, we observed the isotopically labeled glycopeptides consisting of EEQYNSTYR (1189.2 Da) from IgG1 and EEQFNSTFR (1157.2 Da) from IgG2/3 (IgG2 and IgG3 were abbreviated as IgG2/3) (Figure 2). However, glycopeptides with the sequence EEQYNSTFR (1173.2 Da) from IgG3 and EEQFNSTYR (1173.2 Da) from IgG4 were not detected due to their low concentration. The glycopeptides from IgG1 and IgG2/3 could be quantitatively reacted with isotopic reagent, and the obtained glycopeptides by HILIC purification could be quantitatively isolated from mixture for the reason that unglycosylated peptides were not mainly detected (Figure 2A), but Bz- and dBz-labeled glycopeptides with the same peak intensities were observed in the spectra of an equimolar mixture (Figure 2B). These glycopeptides had 10 glycoforms exhibiting a large dynamic range (about 100-fold) with concentration (Supplementary Table S1). Although the glycopeptides of human serum IgG have a small amount of sialic acid [43], loss of sialic acid occurs due to post-source decay during MALDI-TOF MS measurement in positive mode (Supplementary Figures S10). This work was focused on neutral glycopeptides without sialic acid. Also, the results showed that the glycan profiles between IgG1 and IgG2/3 were different (Figure 2C). In MALDI-TOF MS, the MS signal ratio of IgG1 glycopeptides and IgG2/3 glycopeptides depended on the amounts of analytes, but the MS signal ratio of the glycan profile on the same peptide sequence was retained regardless of the concentration of the analytes (Supplementary Figure S6). The difference in ionization efficiency induced by stabilization of the coordination complex (proton adduct ion affected by the proton affinity of the molecules) depended on the concentration and characteristics affected by composition of the molecules. Consequently, this stable isotope labeling of glycopeptides was essential to quantitative glycopeptide profiling by MALDI-TOF MS, which could be used as measurement standard having the same composition and concentration.

### 2.2. Calibration of IgG1 Glycopeptides Using Stable Isotope Labeling

**Figure 3:**
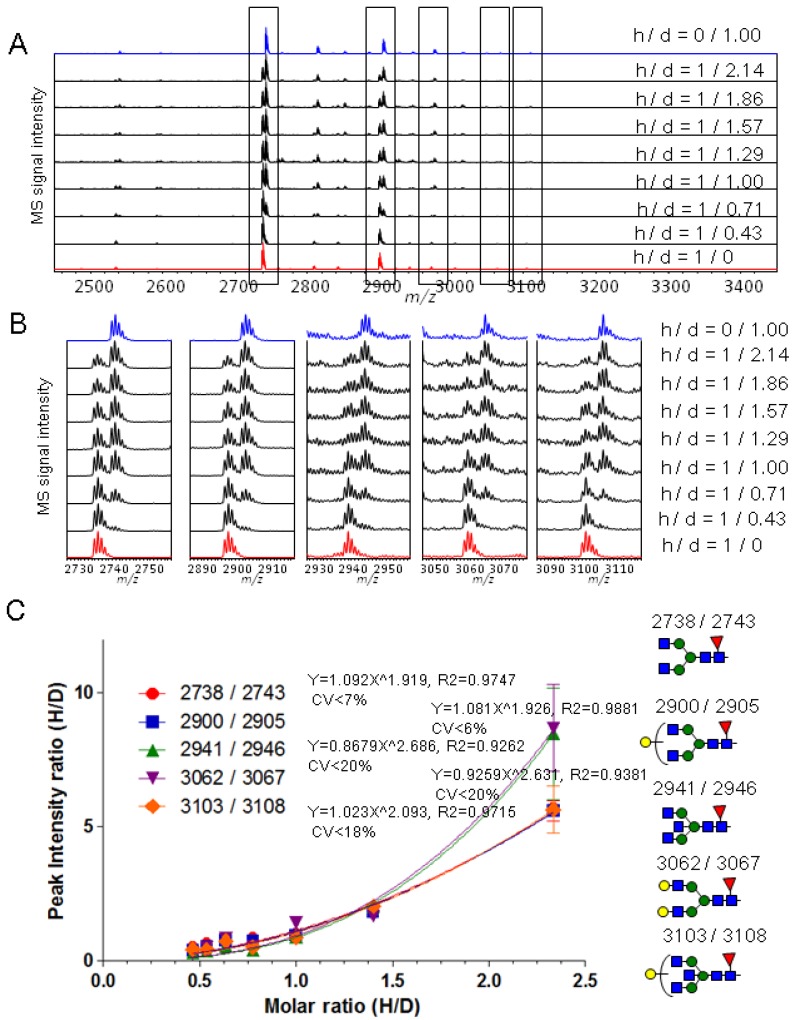
(**A**) MALDI-TOF MS spectra of Bz and d-Bz labeled glycopeptides mixed in different molar ratios (1:0, 1:0.43, 1:0.71, 1:1, 1:1.29, 1:1.57, 1:1.86, 1:2.14, 0:1); (**B**) Enlarged spectra of IgG1 glycopeptides containing Hex_3_HexNAc_4_dHex_1_, Hex_4_HexNAc_4_dHex_1_, Hex_3_HexNAc_5_dHex_1_, Hex_5_HexNAc_4_dHex_1_, Hex_4_HexNAc_5_dHex_1_; (**C**) Calibration curves (peak intensity ratio *vs*. H/D molar ratio, H/D ratio means Bz-/d-Bz-labeled glycopeptides).

To examine the accuracy and reproducibility of glycopeptide quantification using isotopic labeling, we labeled glycopeptides from purified IgG1 with light (Bz) and heavy (d-Bz) reagents, mixed them in different molar ratios (1:0.43, 1:0.71, 1:1, 1:1.29, 1:1.57, 1:1.86, 1:2.14, Bz- to d-Bz-labeled glyco-peptides), and analyzed the mixtures by MALDI-TOF MS (Figure 3). The obtained IgG1 glycopeptides by HILIC purification could be quantitatively isolated from mixture in the same way as human serum IgG experiment. As shown in the spectra of mixture (Figure 3A,B), these spectra exhibited five pairs of peaks, including those with *m/z* 2737.8/2742.8, 2899.8/2904.8, 2940.8/2945.9, 3061.9/3066.9 and 3102.9/3107.9.

These pairs were assigned to the Bz- and d-Bz-labeled glycopeptides containing Hex_3_HexNAc_4_dHex_1_, Hex_4_HexNAc_4_dHex_1_, Hex_3_HexNAc_5_dHex_1_, Hex_5_HexNAc_4_dHex_1_ and Hex_4_HexNAc_5_dHex_1_, respectively. The MS signal intensity ratios of Bz- to d-Bz-labeled glycopeptides were plotted *versus* the expected molar ratios (Figure 3C). The response curves were not quite linear and were fit using power approximation by the approach reported by Anderson *et al*. [45] to provide the R^2^ value and coefficient of variability (CV) of the mean value (n = 3) (Figure 3C). Although the reproducibility of components (*m/z* 2940.8/2945.9 and 3061.9/3066.9) with high CV values present at low concentration was worse than that of components present at high concentration, a resulting response curve of a component was almost overlapped with other component’s response curve (Supplementary Figure S9). Thus, the calibration curves can be used to calculate the concentration of targeted molecules, and also to characterize the glycoform contents.

### 2.3. Quantitative Comparison of Glycopeptides from Human Serum IgG and IgG1 κ from Myeloma Plasma

**Figure 4:**
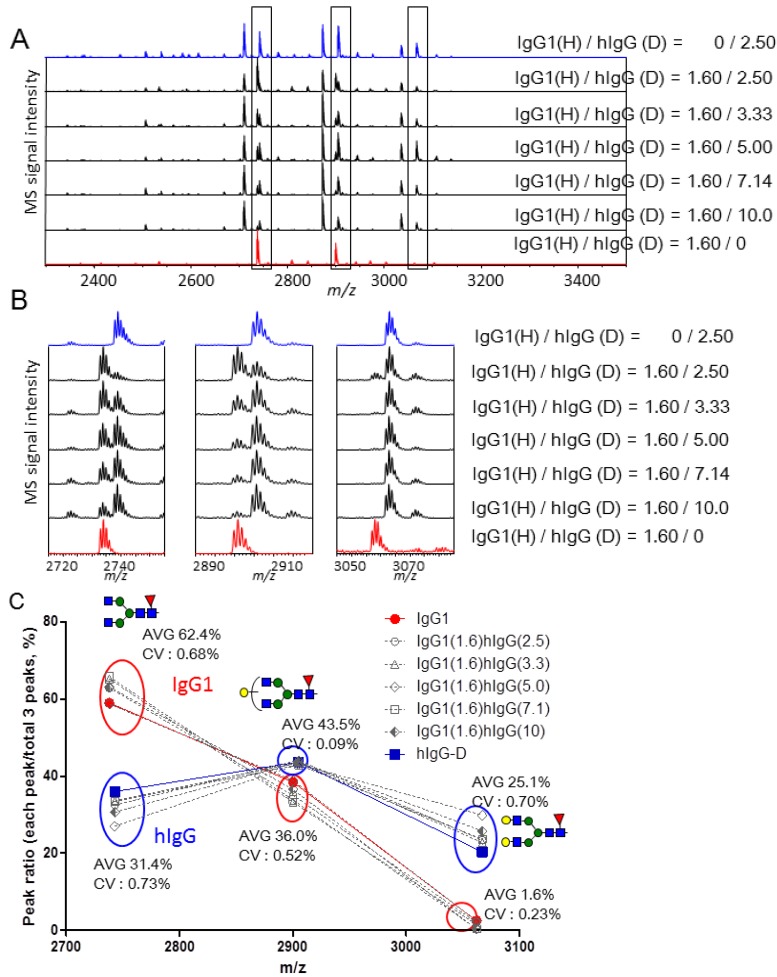
Comparative analysis between IgG1 and hIgG. (**A**) MALDI-TOF MS spectra of different ratio mixtures (0:2.50, 1.60:2.50, 1.60:3.33, 1.60:5.00, 1.60:7.14, 1.60:10.0, 1.60:0 (μg/μg)) of Bz-labeled IgG1 to d-Bz-labeled hIgG glycopeptides; (**B**) Enlarged spectra of IgG1 glycopeptides containing Hex_3_HexNAc_4_dHex_1_, Hex_4_HexNAc_4_dHex_1_, Hex_5_HexNAc_4_dHex_1_; (**C**) The plots of each glycopeptide ratio of the two glycoproteins.

To validate the applicability of the newly developed method to the quantitative glycopeptide profiling of two samples, the relative quantification between IgG1 subclass from human serum IgG and purified IgG1 from human myeloma plasma was performed. The two samples were prepared and labeled with d-BzOSu and BzOSu as described in the experimental Section 3.2 and Section 3.3. The isotopically labeled glycopeptide samples were then mixed in various ratios and analyzed by MALDI-TOF MS (Figure 4).

The different ratio mixtures (0:2.50, 1.60:2.50, 1.60:3.33, 1.60:5.00, 1.60:7.14, 1.60:10.0, 1.60:0 of Bz-labeled IgG1 to d-Bz-labeled hIgG glycopeptides derived from original amounts of protein based on weight (μg)) produced three pairs of peaks. Three pairs were the glycopeptides consisting of three kinds of glycan composition (Hex_3_HexNAc_4_dHex_1_, Hex_4_HexNAc_4_dHex_1_ and Hex_5_HexNAc_4_dHex_1_) and the same amino acid sequence (EEQYNSTYR), so that human serum IgG1 glycopeptides could be identified from the spectra of human serum IgG mixture. The MS signal intensity ratio of each labeled glycopeptide to the total labeled IgG1 glycopeptides containing three kinds of glycans with the same isotope mass tag was retained at each mixture, and also this strategy indicated high accuracy and reproducibility for glycopeptides profiling containing the same amino acid sequence. A difference of glycan profiling between IgG1 among human serum IgG and purified IgG1 was shown by this method (Figure 4C). In addition, the ratio of IgG1 to human serum IgG was approximately 64% (w/w) under the calculation that total amount of IgG1 glycopeptides obtained from 5.00 μg of human serum IgG was twice as much as total amount glycopeptides from 1.60 μg of purified IgG1. This approach showed that isotopic quantitative glycopeptide profiling leads to characterization of glycan profiling and relative quantitation of targeted glycoprotein and glycopeptides in the mixture of biological samples.

### 2.4. Quantitative Monitoring of Glycopeptides for Enzymatic Reaction using Stable Isotope Labeling

#### 2.4.1. Substrate Specificity of Endo-β-*N*-acetylglucosaminidase from Streptococcus Pyogenes (endoS) for hIgG Glycopeptides

**Figure 5:**
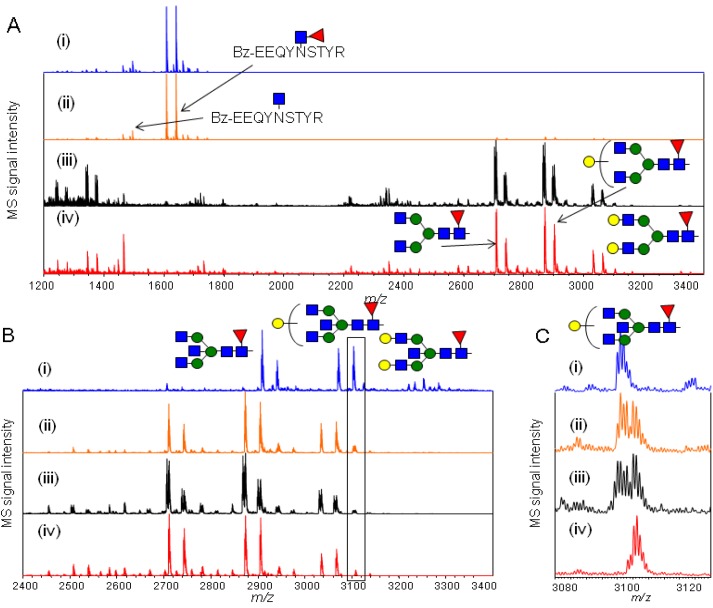
(**A**) MALDI-TOF MS spectra of (i) Bz-labeled hIgG glycopeptides reacted with endoS (ii) equimolar mixture of Bz-labeled hIgG glycopeptides reacted with endoS and d-Bz-labeled hIgG glycopeptides not reacted with endoS (iii) equimolar mixture of Bz-labeled hIgG glycopeptides and d-Bz-labeled hIgG glycopeptides (iv) d-Bz-labeled hIgG glycopeptides; (**B**) Enlarged spectra from *m/z* 2400 to 3400; (**C**) Enlarged spectra of IgG1 glycopeptides containing Hex_4_HexNAc_5_dHex_1_.

To explore the substrate specificity of endo-β-*N*-acetylglucosaminidase from *Streptococcus pyogenes* (endoS) [46], quantitative comparison of the glycopeptides with and without enzymatic reaction was performed. hIgG glycopeptides labeled with BzOSu and d-BzOSu were prepared as described in the experimental Section 3.3 and Section 3.4, then the Bz-labeled glycopeptides were reacted with endoS, and mixed with d-Bz-labeled peptides in one of four ways: (i) Bz-labeled hIgG glycopeptides reacted with endoS; (ii) Bz-labeled hIgG glycopeptides reacted with endoS and d-Bz-labeled hIgG glycopeptides (1:1 ratio) without reaction of endoS; (iii) Bz-labeled hIgG glycopeptides and d-Bz-labeled hIgG glycopeptides in a 1:1 ratio; (iv) d-Bz-labeled hIgG glycopeptides. From MALDI-TOF MS analysis of these samples (Figure 5), it was confirmed that Bz-labeled hIgG glycopeptides and d-Bz-labeled hIgG glycopeptides with the same concentration as an enzymatic substrate could be precisely prepared, and also enhanced ionization potency of labeled glycopeptides allowed for MALDI-TOF MS based facile and quantitative monitoring of enzymatic reaction. The glycopeptides (*m/z* 1464.3 for IgG2/3, 1496.3 for IgG1, and 1610.4 for IgG2/3, 1642.4 for IgG1) bearing a monosaccharide (GlcNAc) or a disaccharide (Fucα1-6GlcNAc) prominently appeared after reaction with endoS (Figure 5A): most of the hIgG glycopeptides were digested by the reaction of endoS. However, glycopeptides containing bisecting *N*-glycans, *i.e.*, containing a GlcNAcβ1-4 linked β-mannose residue in the *N*-glycan core structure, were not digested (Figure 5B). Although Dixon E.V. *et al.* recently reported the susceptibility of endoS for IgG and IgG fragments (Fc and CH2-H, CH2 domain), they did not examine trypsin-digested glycopeptides. After the reaction of endoS, there was a population of uncleaved bisecting biantennary glycans from the CH2-H and CH2 glycoprotein except for IgG and Fc glycoprotein which can be recognized by carbohydrate binding modules (CBM) on endoS. Thus, the result is consistent with our result that endoS is hard to cleave biantennary *N*-glycans containing bisecting GlcNAc in comparison with other biantennary N-glycans on IgG glycopeptides [46,47].

#### 2.4.2. Characterization of the hIgG Glycopeptide Isoforms by endo-β-*N*-Acetylglucosaminidase from *Streptococcus pneumoniae* (endo-D) Combined with exo-β-*N*-Acetylglucosaminidase (β-GlcNAc’ase)

**Figure 6:**
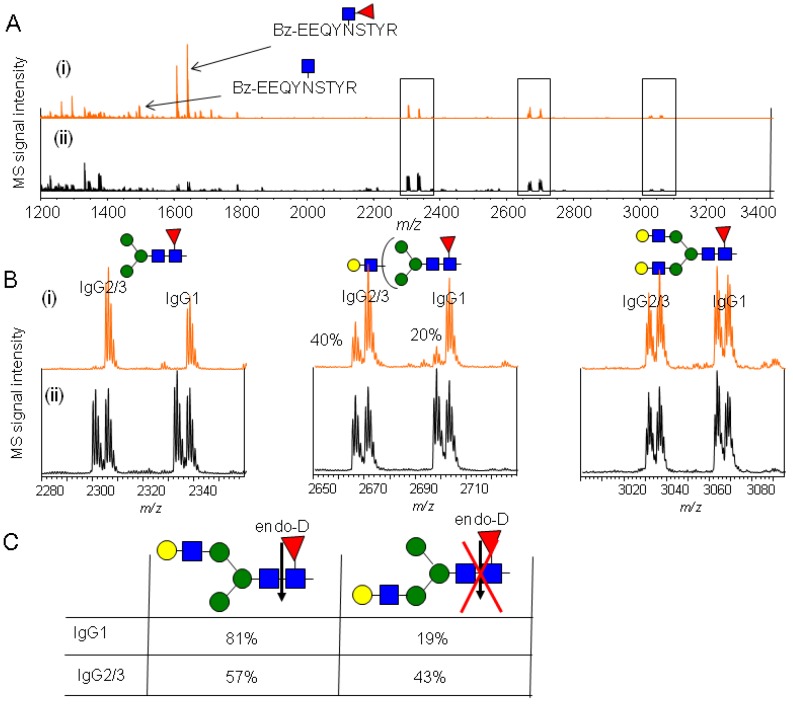
(**A**) MALDI-TOF MS spectra of (i) an equimolar mixture of Bz-labeled hIgG glycopeptides reacted with both β-GlcNAc’ase and endo-D, and d-Bz-labeled hIgG glycopeptides reacted with only β-GlcNAc’ase (ii) an equimolar mixture of Bz-labeled hIgG glycopeptides reacted with only β-GlcNAc’ase and d-Bz-labeled hIgG glycopeptides reacted with only β-GlcNAc’ase; (**B**) Enlarged spectra of glycopeptides containing Hex_3_HexNAc_2_dHex_1_, Hex_4_HexNAc_3_dHex_1_, Hex_5_HexNAc_4_dHex_1_; (**C**) The molar ratio of glycoforms on the IgG1 and IgG2/3 glycopeptides.

To characterize the glycan structural isomers of the hIgG glycopeptides by MALDI-TOF MS analysis, quantitative comparison of glycopeptides was performed using the known substrate specificity of exo-β1–2,3,4,6-*N*-acetylglucosaminidase (β-GlcNAc’ase) and endo-β-*N*-acetyl-glucosaminidase from *Streptococcus pneumoniae* (endo-D). hIgG glycopeptides labeled with BzOSu and d-BzOSu were prepared and digested with β-GlcNAc’ase as described in the experimental Section 3.6. Both Bz- and d-Bz-labeled glycopeptides from hIgG were converted to glycopeptides with an unsubstituted α-mannosyl residue at the terminal mannose of the *N*-glycan core structure in part, and then these labeled glycopeptides were prepared as a mixture containing the upper branch and the lower branch with galactosylated extension on the biantennary glycan. The *N*-glycans from normal hIgG have previously been identified by HPLC separation [48,49]. The molar ratio of the upper to the lower galactosylated extension on partially truncated biantennary glycans with core fucosylation was shown to be about 2.5:1.0. Generally, glycan structural isomers with the same molecular weight cannot be determined by mass spectrometry, but can be recognized by a specific protein (lectin) or enzyme exhibiting high affinity and substrate specificity. In this experiment, quantitative characterization of glycan isomers on glycopeptide profiling was accomplished using stable isotope labeling and the high substrate specificity of endo-D. Endo-D cleaves only *N*-glycans with an unsubstituted α-mannosyl residue at the C-3 position of the terminal mannose of the *N*-glycan core structure from glycopeptides [50]. These two approaches allowed determination of the glycan structural isomer. hIgG glycopeptides labeled with BzOSu were reacted with β-GlcNAc’ase and then with endo-D, and then mixed with d-Bz-labeled hIgG in one of two ways: (i) Bz-labeled hIgG glycopeptides were reacted with both β-GlcNAc’ase and endo-D, whereas d-Bz-labeled hIgG glycopeptides were reacted with only β-GlcNAc’ase; (ii) Bz-labeled hIgG glycopeptides were reacted with only β-GlcNAc’ase and d-Bz labeled hIgG glycopeptides were reacted with only β-GlcNAc’ase. It was confirmed that Bz-labeled hIgG glycopeptides and d-Bz-labeled hIgG glycopeptides with the same concentration was precisely prepared, and then enhanced ionization of labeled glycopeptides allowed for MALDI-TOF MS based facile and quantitative characterization of glycan isomers on glycopeptides (Figure 6). The glycopeptides (*m/z* 1464.3 for IgG2/3, 1496.3 for IgG1, and 1610.4 for IgG2/3, 1642.4 for IgG1) bearing a monosaccharide (GlcNAc) or a disaccharide (Fucα1-6GlcNAc) appeared after reaction with endo-D (Figure 6A). The MS signals of these digested glycopeptides were prominent because most of the glycopeptides had reacted with endo-D. All hIgG glycopeptides (*m/z* 2299.6 for IgG2/3, 2331.6 for IgG1 consisting of Hex_3_HexNAc_2_dHex_1_) with unsubstituted α-mannosyl residues in the trimannosyl *N*-glycan core structure were completely digested with endo-D, but glycopeptides (*m/z* 3029.7 for IgG2/3, 3061.7 for IgG1 consisting of Hex_5_HexNAc_4_dHex_1_) containing fully substituted α-mannosyl residues remained undigested. In the case of glycopeptides (*m/z* 2664.7 for IgG2/3, 2696.7 for IgG1 consisting of Hex_4_HexNAc_3_dHex_1_) containing partially substituted α-mannosyl residues, 40% and 20% of the MS signal remained, respectively (Figure 6B). Calculations based on the calibration curves for the target molecules (Supplementary Figure S9) showed that the molar ratios of the upper to lower galactosylated extension were 81:19 for IgG1 glycopeptides and 57:43 for IgG2/3 glycopeptides (Figure 6C). Finally, it was found that the ratio of the *N*-glycan structural isomer containing the galactosylated extension was different between IgG1 and IgG2/3 from human serum IgG. Furthermore, isotopic quantitative glycopeptide profiling by MALDI-TOF MS was useful for characterizing glycan structural isomers by combination with the highly specific recognition of an enzyme such as endo-D.

## 3. Experimental Section

### 3.1. General Information

The ^1^H-, ^13^C-NMR spectra were obtained on a JEOL JNM ECA instrument (JEOL, Tokyo, JAPAN) at 600 MHz for ^1^H-NMR and 150 MHz for ^13^C-NMR. Deuterium-labeled benzoic acid was purchased from Cambridge Isotope Laboratories (Andover, MA, USA), and benzoic acid was purchased from Tokyo Chemical Industry (Tokyo, Japan). Human serum immunoglobulin G (hIgG), IgG1, kappa from human myeloma plasma and Sepharose 4B were purchased from Sigma-Aldrich (Milwaukee, WI, USA). RapiGest SF was purchased from Waters (Milford, MA, USA). TPCK trypsin was purchased from Thermo Fisher Scientific (Rockford, IL, USA). Sephadex G-25 was purchased from GE Healthcare Life Sciences (Uppsala, Sweden). Endo-β-*N*-acetylglucosaminidase from *Streptococcus pyogenes* (endoS) was overproduced in *E. coli* using a previously reported procedure [46]. Endo-β-*N*-acetylglucosaminidase from *Streptococcus pneumoniae* (endo-D) was purchased from New England Biolabs (Ipswich, MA, USA). β1–2,3,4,6-*N*-Acetylglucosaminidase from *Streptococcus pneumoniae* was purchased from Merck KGaA (Darmstadt, Germany). Other chemicals were purchased from Wako Pure Chemical Industries (Osaka, Japan).

### 3.2. Synthesis of Benzoic Acid N-Succinimidyl Ester (Bz and d-Bz Labeling Reagents)

Benzoic acid (ring d-5, heavy isotope, d-Bz) (127 mg, 1.00 mmol) or benzoic acid (ring d-0, light isotope, Bz) (122 mg, 1.00 mmol), *N*-hydroxysuccinimide (230 mg, 2.00 mmol) and triethylamine (167 μL, 1.20 mmol) were dissolved in anhydrous dimethylformamide (DMF) (1 mL). After addition of dicyclohexylcarbodiimide (DCC) (206 mg, 1.20 mmol), the solution was stirred at room temperature for 3 h. The reaction mixture was filtered to remove the precipitate, and concentrated *in vacuo*. The resulting syrup was crystallized from isopropanol (5 mL) to yield benzoic acid *N*-succinimidyl ester (d-Bz labeling reagent, 184 mg, 82%) (Bz labeling reagent, 170 mg, 78%). R_f_ = 0.42 (toluene-EtOAc = 10:1); ^1^H-NMR (600 MHz, CDCl_3_): δ 2.91 (s, 4H) for the heavy isotope, δ 2.91 (s, 4H), 7.52 (dd, 2H), 7.69 (dd, 1H), 8.14 (dd, 2H) for the light isotope; ^13^C-NMR (125 MHz, CDCl_3_): δ 25.63, 124.88, 128.31, 130.13, 134.38, 161.82, 169.24 for the heavy isotope, δ 25.65, 125.08, 128.83, 130.55, 134.91, 161.84, 169.23 for the light isotope.

### 3.3. Preparation of hIgG and IgG1 Glycopeptides

Human IgG (200 μg) or IgG1 (200 μg) was dissolved in 500 μL of 50 mM ammonium bicarbonate solution and heated at 100 °C for 15 min. After the sample cooled, 50 μL of 1% aqueous RapiGest SF (v/v) and 2 μL of TPCK-treated trypsin (20 μg) were added, followed by incubation at 37 °C for 300 min. The sample was heated at 100 °C for 15 min to inactivate the enzyme, desalted by passage through a G-25 gel filtration column (0.8 × 3.5 cm), and concentrated using a centrifugal evaporation system.

### 3.4. Stable Isotope Labeling of Glycopeptides

The glycopeptides were dissolved in 20 μL of water, then 10 μL of pyridine and 20 μL of 200 mM benzoic acid *N*-succinimidyl ester solution (d-Bz or Bz isotope labeling reagent) in DMF was added and reacted at 57 °C for 12 h. After addition of 60 μL of 0.5 M NaOH_aq_ to de-esterify the product, the sample was mixed on a vortex mixer at room temperature for 30 min. Water (200 μL) was added, the sample was washed with EtOAc (400 μL × 3 times) to remove excess reagent, and the aqueous layer was collected and concentrated using a centrifugal evaporation system. The sample was desalted on a C-18 Spin column (20 mg of C-18 reverse phase silica gel) and concentrated using a centrifugal evaporation system. Further purification was done by HILIC method, which was modified Wada’s [13]. The sample was dissolved in 20 μL of water, added to a mixture of Sepharose 4B (wet vol. 50 μL), 100 μL of ethanol and 400 μL of butanol, then mixed on a tube rotator at room temperature for 1 h. The resin was washed thoroughly with 2 ml of 10:2.5:2.2:0.3 (v/v/v/v) butanol/ethanol/water/formic acid to remove unglycosylated peptides, then the labeled glycopeptides were eluted with 1.3 mL of 25% ethanol and concentrated using a centrifugal evaporation system.

### 3.5. Enzymatic Reaction of Bz-Labeled Glycopeptides with EndoS

The Bz-labeled glycopeptides obtained from 50 μg of hIgG were dissolved in 5 μL of 10 mM sodium phosphate buffer (pH 7.4) and incubated with endoS (0.5 μL, 1.0 μg/μL) at 37 °C for 12 h. The sample was directly analyzed by MALDI-TOF MS without purification.

### 3.6. Enzymatic Reaction of Labeled Glycopeptides by β-N-Acetylglucosaminidase (β-GlcNAc’ase)

The Bz- or d-Bz-labeled glycopeptides obtained from 100 μg of hIgG were dissolved in 10 μL of 50 mM sodium acetate buffer (pH 5.0) and incubated with β-GlcNAc’ase (50 mU, 2 μL) at 37 °C for 12 h. After heating at 100 °C for 15 min, the sample was desalted on a C-18 Spin column (20 mg of C-18 resin) and concentrated using a centrifugal evaporation system.

### 3.7. Enzymatic Reaction of Bz-Labeled Glycopeptides by Endo-D

The hydrolyzed Bz-labeled glycopeptides obtained from 50 μg of hIgG were dissolved in 5 μL of 10 mM sodium phosphate buffer (pH 7.4), and incubated with endo-D (0.5 μL, 25 units) at 37 °C for 12 h. The sample was directly analyzed with MALDI- TOF MS without purification.

### 3.8. MALDI Sample Preparation and Measurement

The sample solution (0.5~1.0 μL) was mixed with 1 μL of matrix (10 mg/mL 2,5-dihydroxybenzoic acid solution [Shimadzu Biotech, Kyoto, Japan]) on a target plate. Mass spectra were acquired in positive ion mode using a matrix assisted laser desorption ionization quadrupole ion trap time of flight mass spectrometer (MALDI-QIT-TOF MS) (AXIMA Resonance, Shimadzu Biotech, Manchester, UK). Ions were generated by a pulsed nitrogen UV laser (337 nm, 5 Hz).

## 4. Conclusions

A novel strategy based on stable isotope labeling with BzOSu and d-BzOSu allows the highly sensitive relative quantitation of glycopeptides. To our knowledge, this approach is the first demonstration of isotopic reagents for glycopeptide labeling allowing comparative glycopeptide profiling studies by MALDI-TOF MS. We synthesized Bz and d-Bz reagents, determined the optimized reaction conditions for Bz and d-Bz labeling, and validated the detection sensitivity of benzoyl labeling using egg yolk glycopeptides, human IgG, and bovine ribonuclease B glycopeptides as model glycopeptides. The sensitivity of detection of the glycopeptides was approximately 2 fmol (Supplementary Figure S6). The high reproducibility and accuracy of the method was confirmed using glycopeptides digested from hIgG. Two examples were used to demonstrate the applicability of the procedure: glycopeptides digested from human serum IgG and purified myeloma plasma IgG1 were quantitatively compared by MALDI-TOF MS analysis, and the glycopeptides from hIgG were compared with and without enzymatic treatment. This labeling approach has two important advantages including (i) higher sensitivity than non-labeled glycopeptides in both positive and negative modes (Supplementary Figures S1–S3); and (ii) MS/MS profiles arising from X-, Y-, A-, B-type fragmentation enable rapid structural identification in both positive and negative modes (Supplementary Figure S8). Although this study focused on the analysis of *N*-linked glycopeptides, the strategy can be directly applied to other types of glycopeptides, such as *O*-linked glycopeptides. In addition to Bz and d-Bz labeling, other isotopic reagents are compatible with quantitative glycoproteomics strategies. Furthermore, isotopic labeling of glycopeptides mixtures derived from biological samples such as serum will be applicable to LC-MALDI TOF MS and LC-ESI-MS analysis, allowing high separation performance. This strategy therefore represents a facile and versatile analytical methodology for comparative glycoproteomics and holds promise for biological analyses.

## Supplementary Materials

Supplementary materials can be accessed at: http://www.mdpi.com/1420-3049/19/7/9944/s1.
