# Supplementary material for: Relative Quantitation of Glycopeptides Based on Stable Isotope Labeling Using MALDI-TOF MS
Source: Molecules. 2014 Jul 9;19(7):9944–61. doi: 10.3390/molecules19079944 (PMC6271863; doi:10.3390/molecules19079944)
Supplement: Supplementary file 1 [file molecules-19-09944-s001.pdf]

Supplementary Materials

Material and Methods

For investigation of labeling reagents, we prepared the glycopeptide from egg yolk sialoglycopeptide (Tokyo Chemical Industry, Tokyo, Japan). Sialoglycopeptide (10 μM, 100 μL) was mixed with 110 μL of 7 M ammonium hydroxide solution, and reacted with *O*-methylisourea hydrochloride (9 M, 30 μL) at 65 °C for 30 min. using the method reported by Beardsley [1]. After addition of 300 μL of 10% trifluoroacetic acid, the solution was concentrated using a centrifugal evaporation system. The sample was dissolved in 100 μL of 0.8% trifluoroacetic acid and heated at 90 °C for 30 min to remove sialic acid by acid-hydrolysis. The product was isolated using a graphite carbon column (InertSep GC 50 mg, GL Science, Tokyo, Japan). H-Homoarg-Val-Ala-Asn ($Hex_5HexNAc_4$)-Homoarg-Thr-OH containing single amino group at the *N*-terminal end of glycopeptides, was obtained. Labeled glycopeptides were prepared as the described in experimental section. Acetyl, naphtoyl, pyrenoyl reagents were prepared from acetic acid, 2-naphthoic acid, 1-pyrenecarboxylic acid. 10 pmol of each samples was analyzed using MALDI-TOF MS.

For preparation of glycopeptides consisted of *N*LTK peptide sequence, bovine ribonuclease B (200 μg, Sigma-Aldrich, Milwaukee, WI, USA) was dissolved in 500 μL of 50 mM ammonium bicarbonate solution, and heated at 100 °C for 15 min. After the sample cooled, 50 μL of 1% aqueous RapiGest SF (v/v) and 2 μL of TPCK-treated trypsin (20 g) were added, followed by incubation at 37 °C for 300 min. The sample was heated at 100 °C for 15 min, and then desalted by G-25 gel filtration column (0.8 × 3.5 cm) and concentrated using a centrifugal evaporation system. The sample was dissolved in 20 μL of water, added with Sepharose 4B (wet vol. 50 μL), 100 μL of ethanol, 400 μL of buthanol, and mixed with tube rotator at room temperature for 1 h. The resin was washed thoroughly with 2 mL of 10:2.5:2.2:0.3 (v/v/v/v) buthanol/ethanol/ water/formic acid, the labeled glycopeptides was eluted with 1.3 mL of 25% ethanol, concentrated using a centrifugal evaporation system. Sample (100 μL) was mixed with 110 μL of 7 M ammonium hydroxide solution, and reacted with *O*-methylisourea hydrochloride (9 M, 30 μL) at 65 °C for 30 min. After addition of 300 μL of 10% trifluoroacetic acid, the solution was concentrated using a centrifugal evaporation system. Labeled glycopeptides were prepared as the described in the Experimental Section.

Figure S1. MS spectra of benzoyl (black triangle) and acetyl (gray triangle) and unlabeled (white triangle) glycopeptides.

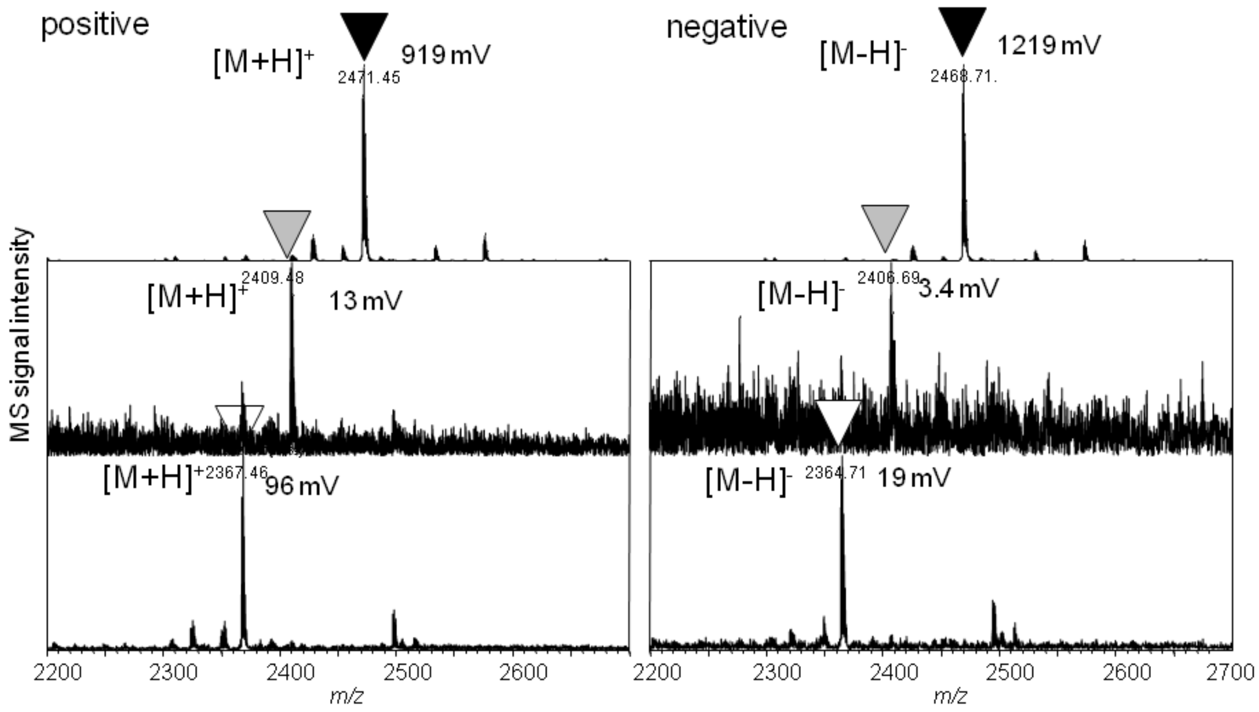

Figure S2. MS spectra of a 1:1 mixture of unlabeled and benzoyl glycopeptides from hIgG in positive and negative mode.

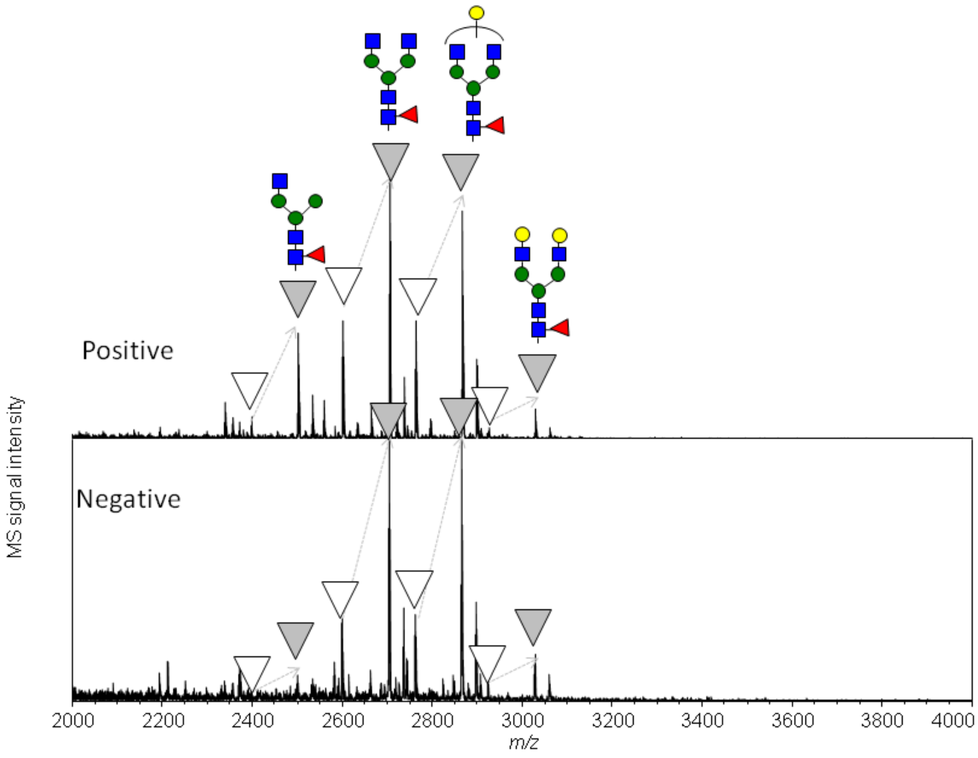

Figure S3. MS spectra of a 1:1 mixture of unlabeled and benzoyl glycopeptides from bovine RN’ase B in positive and negative mode.

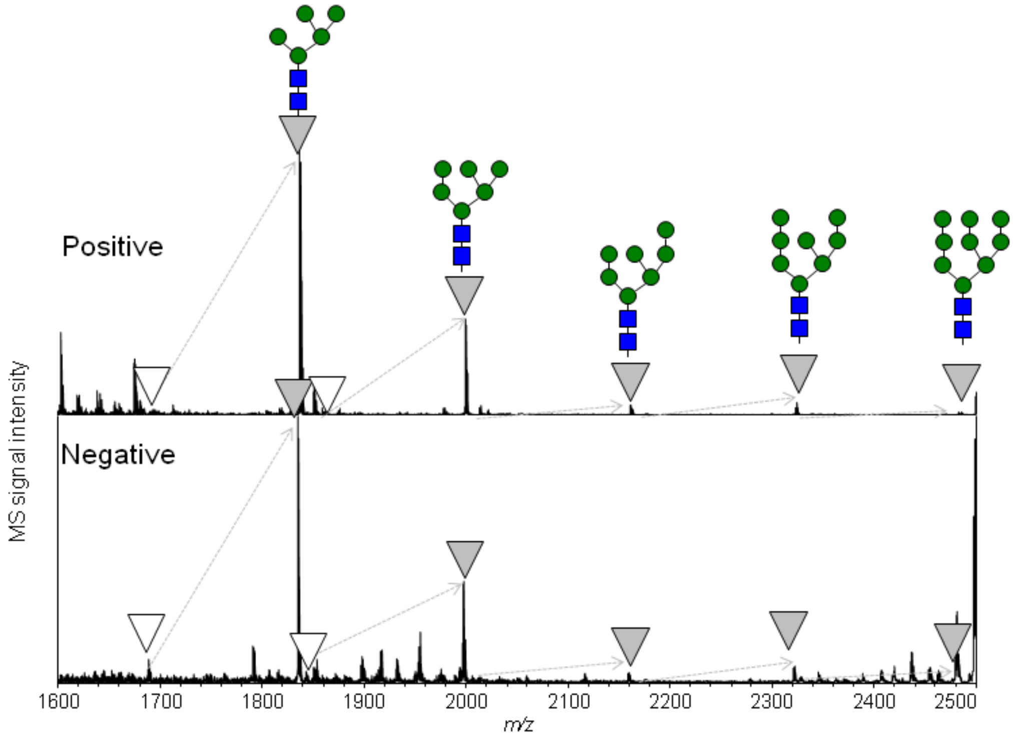

Figure S4. MS spectra of a 1:1 mixture of benzoyl and naphthoyl glycopeptides from egg yolk glycopeptides in positive and negative mode

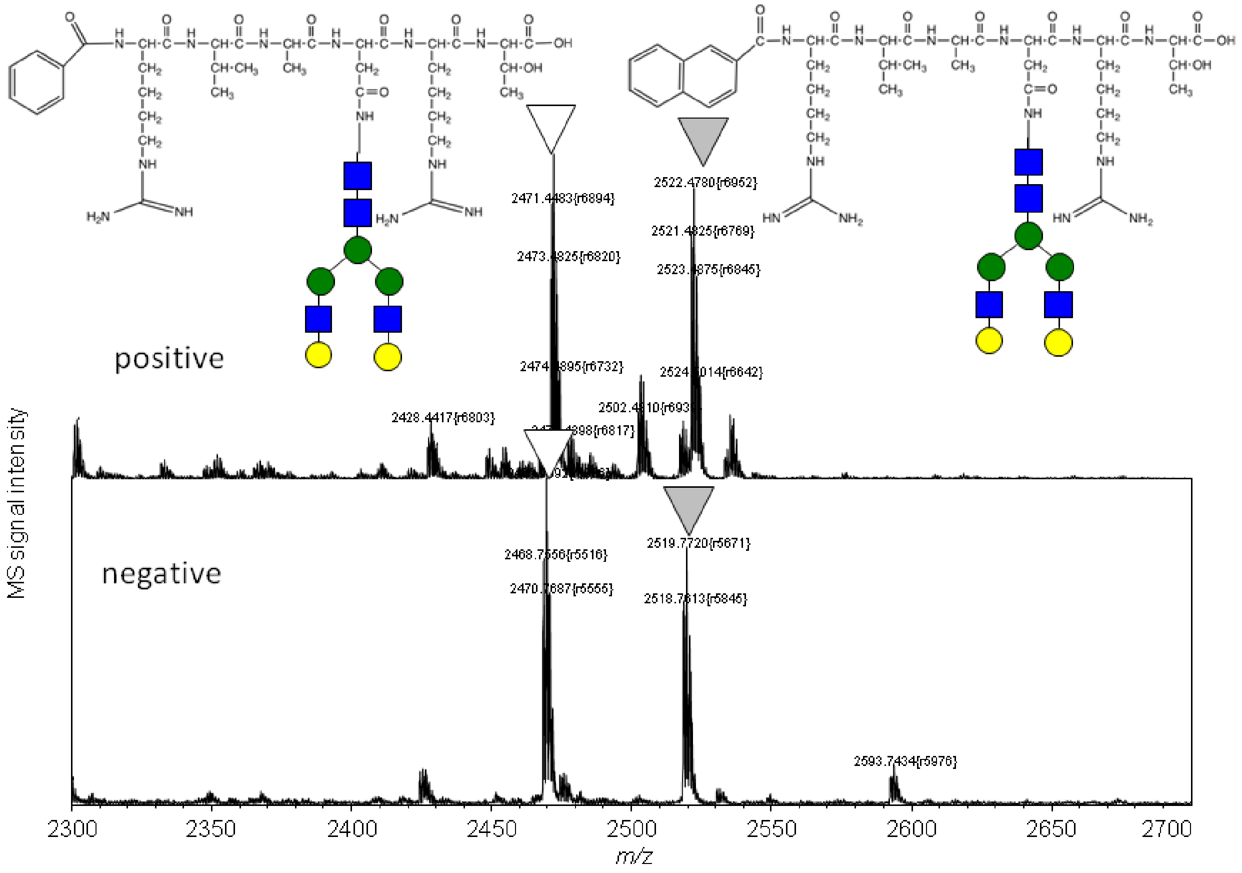

Figure S5. MS spectra of a 1:1 mixture of benzoyl and pyrenoyl glycopeptides from egg yolk glycopeptides in positive and negative mode.

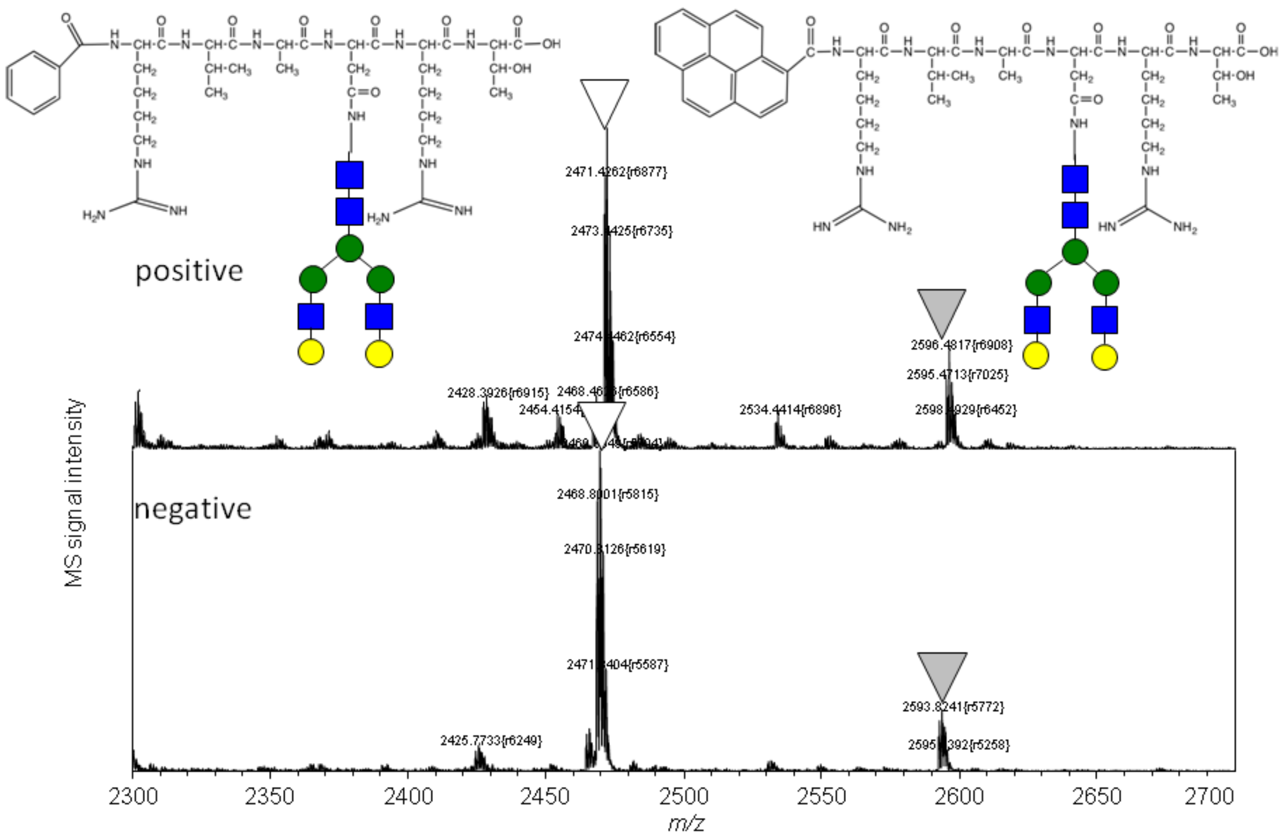

Figure S6. (**A**) MS spectra of a 1:1 mixture of Bz- and d-Bz-labeled glycopeptides from hIgG using different amounts (20, 4, 2, 1.2, 0.4 pmol) of sample in positive mode; (**B**) The plot of each peak ratio of 6 isotopic pairs; (**C**) The plot of each peak ratio of 3 isotopic pairs from the same peptides.

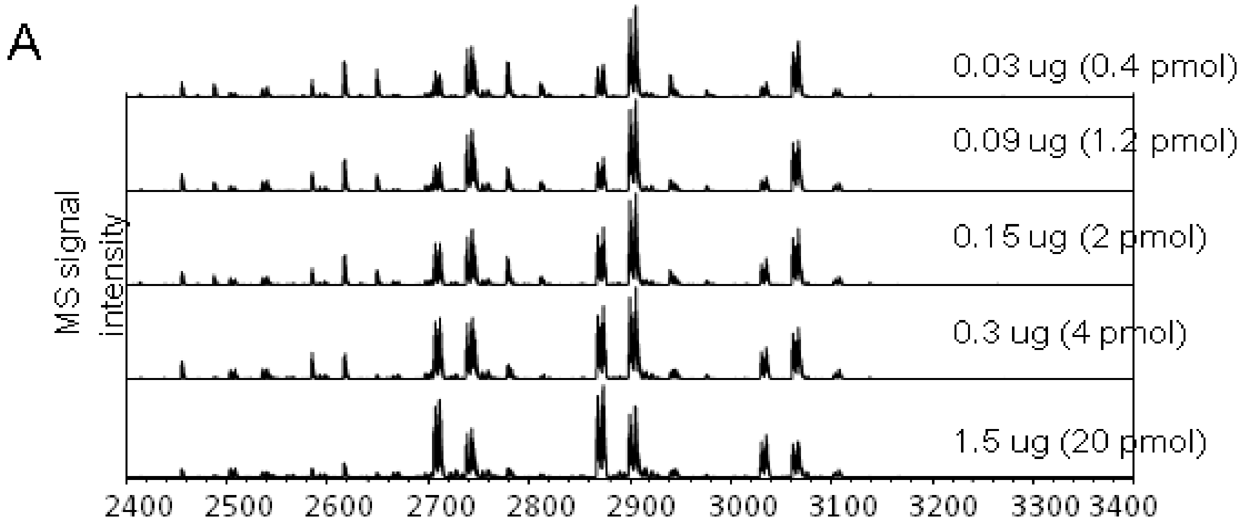

Figure S6. *Cont.*

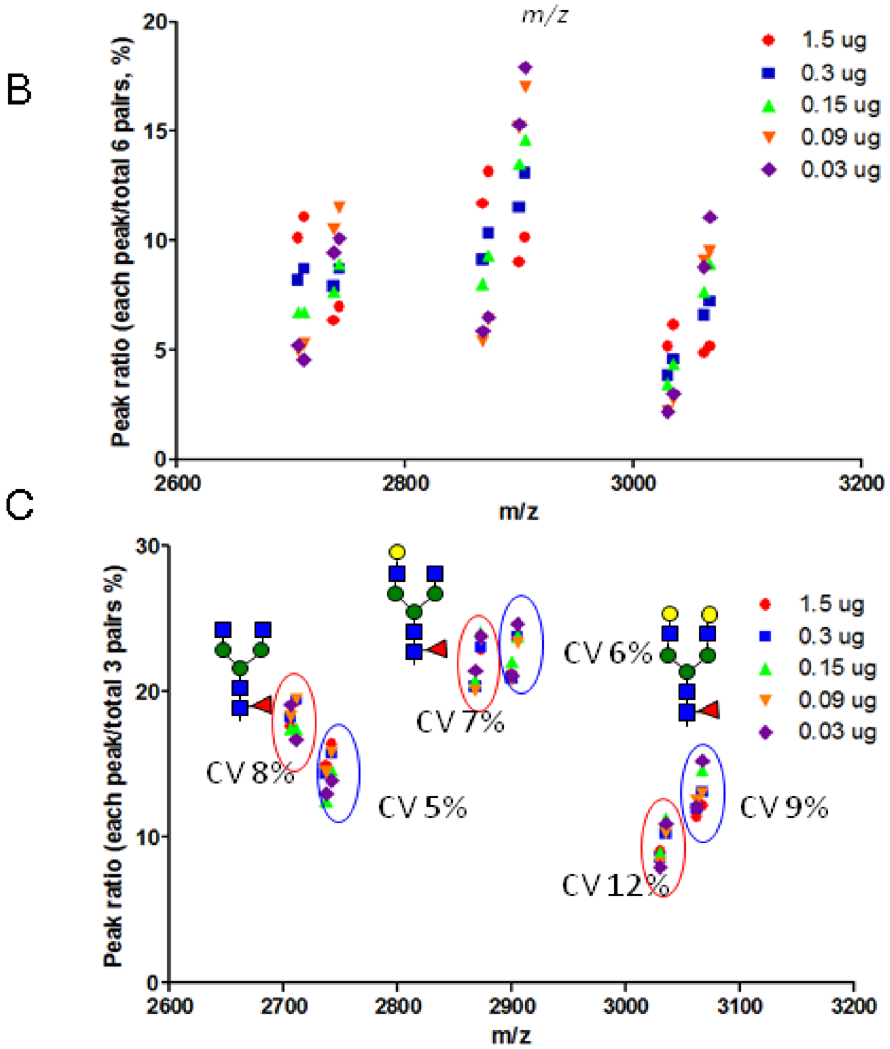

Table S1. The data from Figure 2 (1:1 mixture of Bz- and d-Bz-labeled glycopeptides from hIgG). (Values highlighted in yellow are estimated values because the targeted peak overlapped with other peaks.)

				IgG1 Bz-EEQYNSTYR (1293.23)			IgG2 Bz-EEQFNSTFR (1261.23)		
**No.**	**Composition (Hex, HexNAc, dHex) Strcture**	**Glycan (mass)**	**Bz**	**calcd [M+H]+**	**Found (*m/z*)**	**Intensity (mV)**	**calcd [M+H]+**	**Found (*m/z*)**	**Intensity (mV)**
1	3 3 1 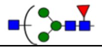	1241.45	H	2534.68	2534.57	13	2502.68	2502.54	25
			D	2539.71	2539.57	13	2507.71	2507.58	26
2	3 4 0 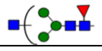	1298.47	H	2591.7	2591.61	9.1	2559.7	2559.63	8.7
			D	2596.73	2596.64	7.1	2564.73	2564.84	8.3
3	4 3 1 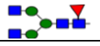	1403.5	H	2696.73	2696.74	8.4	2664.73	2664.71	15
			D	2701.76	2701.66	9.3	2669.76	2669.72	15
4	3 4 1 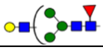	1444.53	H	2737.76	2737.7	69	2705.76	2705.77	151
			D	2742.79	2742.71	65	2710.79	2710.72	145
5	4 4 0 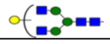	1460.52	H	2753.75	2753.75	12	2721.75	2721.64	12
			D	2758.78	2758.74	11	2726.78	2726.72	13
6	4 4 1 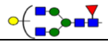	1606.58	H	2899.81	2899.74	95	2867.81	2867.77	159
			D	2904.84	2904.86	95	2872.84	2872.72	165
7	3 5 1 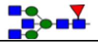	1647.61	H	2940.84	2938.75	20	2908.84	2908.8	14.6
			D	2945.87	2943.96	24	2913.87	2913.8	18
8	5 4 1 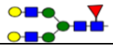	1768.63	H	3061.86	3061.72	31	3029.86	3029.71	65
			D	3066.89	3066.74	36	3034.89	3034.77	67
9	4 5 1 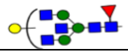	1809.66	H	3102.89	3102.86	10	3070.89	3070.75	6.7
			D	3107.92	3107.74	12	3075.92	3075.86	8.1
10	5 5 1 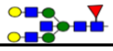	1971.71	H	3264.94	3264.9	1.1	3232.94	3232.67	1
			D	3269.97	3269.8	1	3237.97	3237.85	1.5

Figure S7. MS/MS spectra of Bz and d-Bz-labeled glycopeptides from hIgG in positive mode.

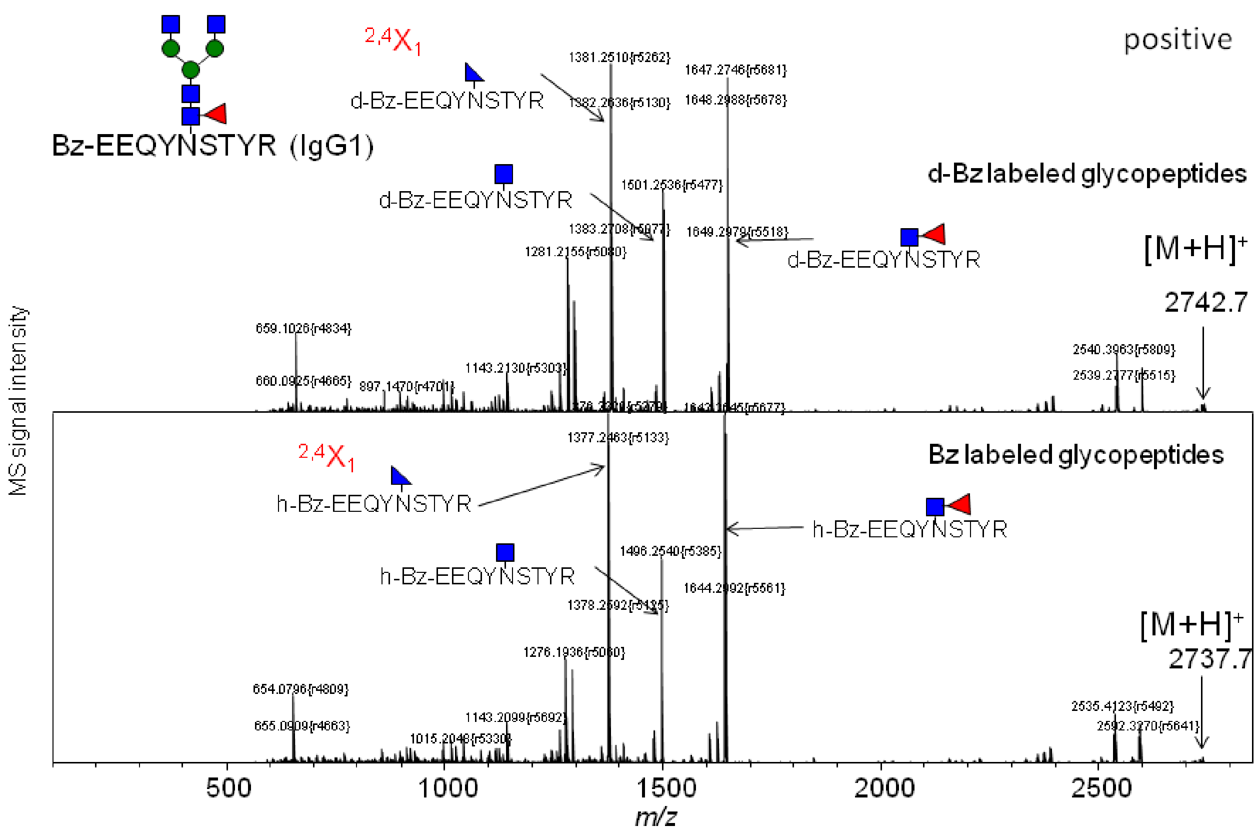

Figure S8. MS/MS spectra of Bz and d-Bz-labeled glycopeptides from hIgG in negative mode.

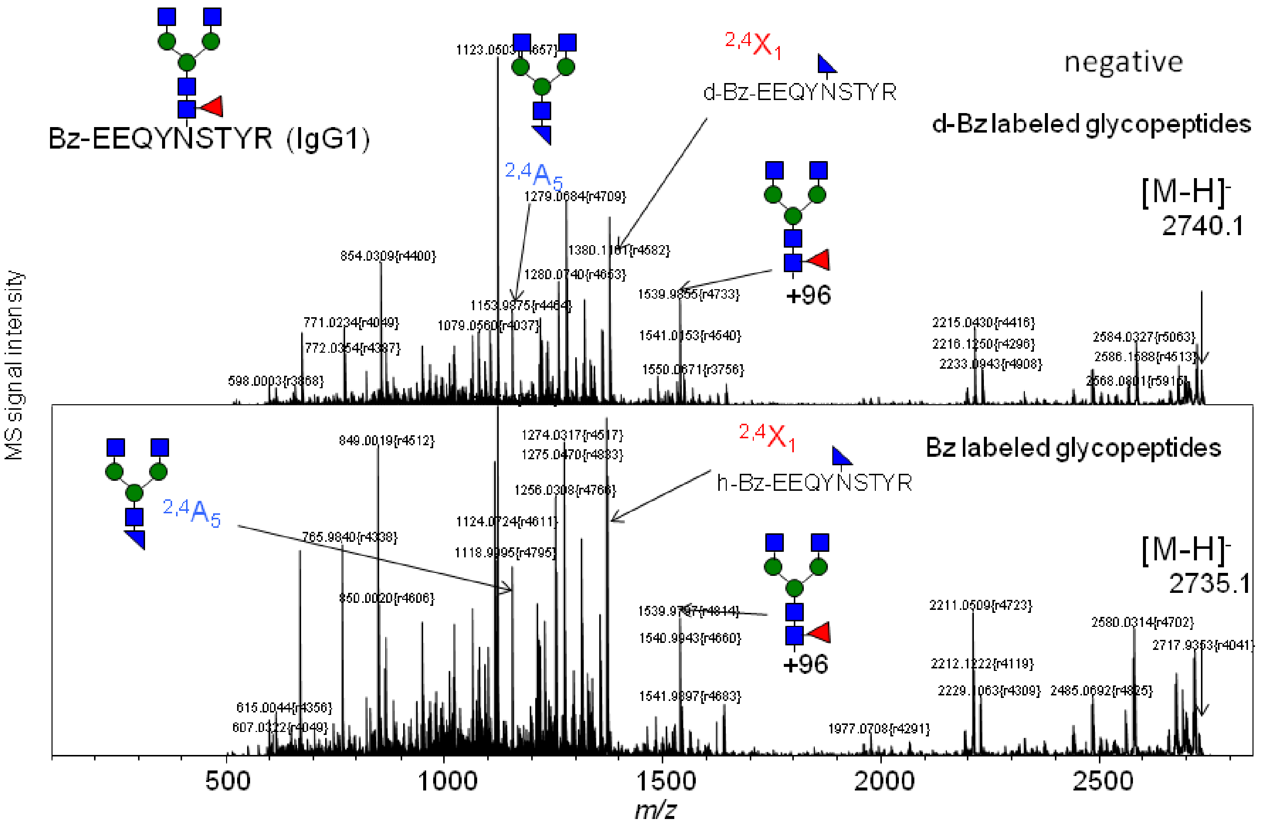

Figure S9. Calibration of glycopeptides from hIgG after reaction with β-GlcNAc'ase (**A**) MS spectra of Bz- and d-Bz-labeled glycopeptides mixed in different molar ratios (0.1:1, 0.3:1, 0.75:1, 1:1, 1.5:1, 2:1); (**B**) Calibration curves (peak intensity ratio vs. molar ratio).

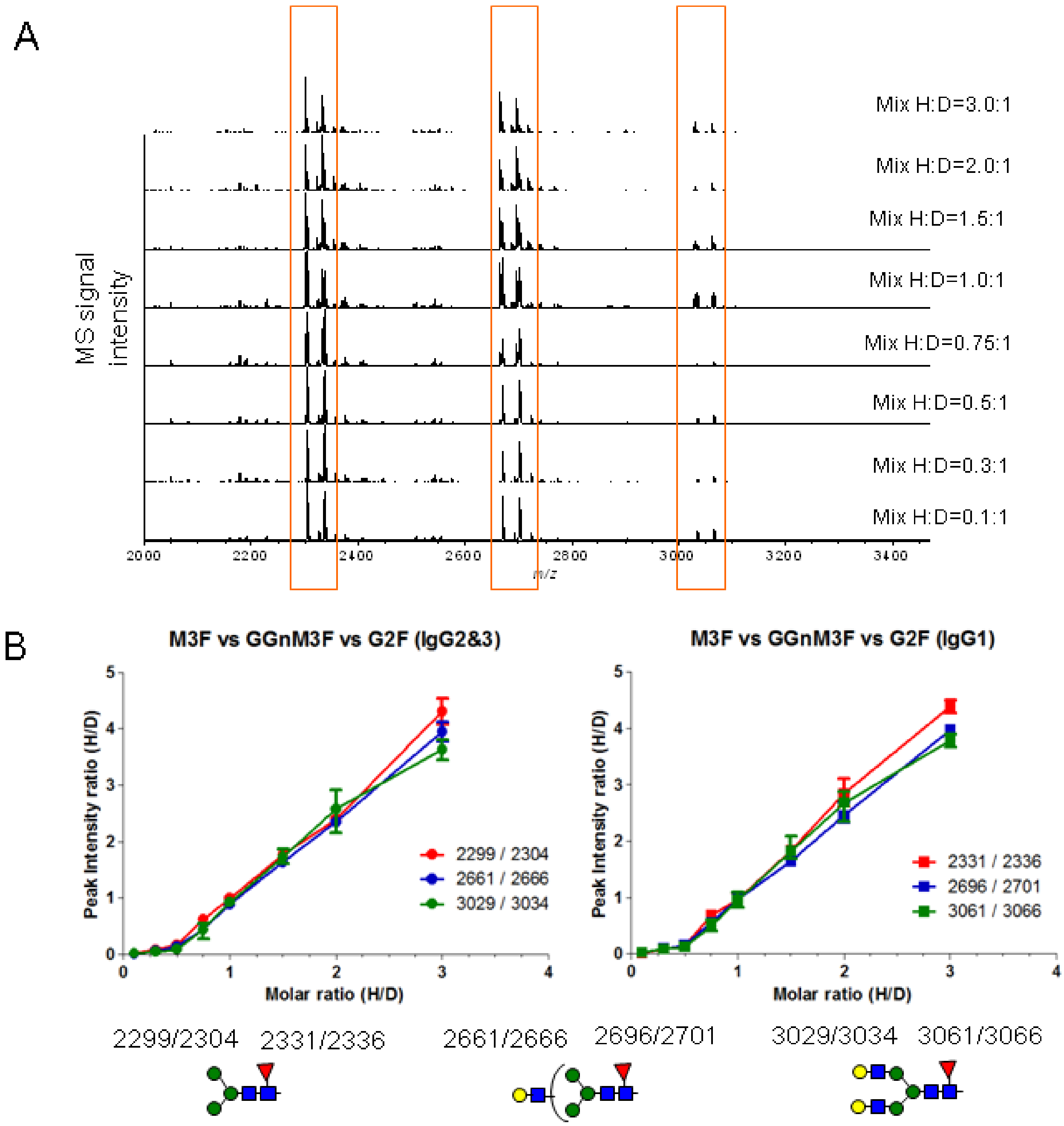

Figure S10. MS spectra of a 1:1 mixture of Bz- and d-Bz-labeled glycopeptides from hIgG in positive mode (**lower**) and negative mode (**upper**).

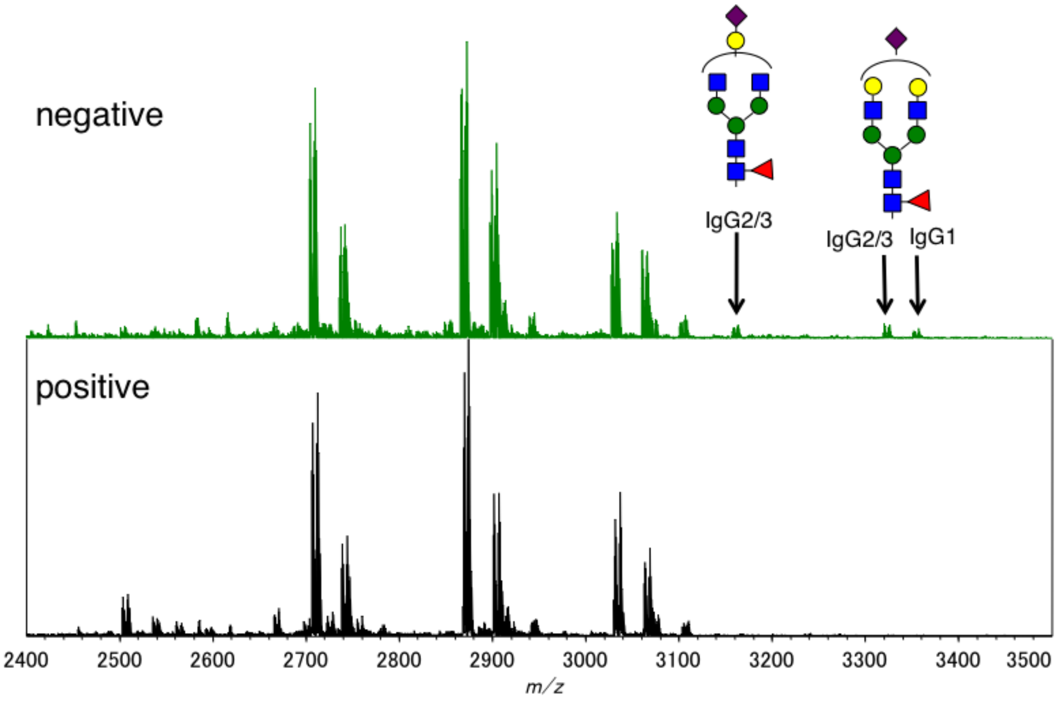

Reference

1. Beardsley, R.L.; Reilly, J.P. Optimization of guanidination procedures for MALDI mass mapping. *Anal. Chem.* **2002**, *74*, 1884–1890.
